# Evaluating the effectiveness of intensive versus non‐intensive image interpretation education for radiographers: a randomised control trial study protocol

**DOI:** 10.1002/jmrs.264

**Published:** 2018-02-01

**Authors:** Michael J. Neep, Tom Steffens, Patrick Eastgate, Steven M. McPhail

**Affiliations:** ^1^ Department of Medical Imaging Logan Hospital Meadowbrook Queensland Australia; ^2^ Centre for Functioning and Health Research Metro South Health Brisbane Australia; ^3^ School of Public Health & Social Work Queensland University of Technology Kelvin Grove, Brisbane Australia; ^4^ Institute of Health and Biomedical Innovation Queensland University of Technology Kelvin Grove, Brisbane Australia; ^5^ Department of Medical Imaging Princess Alexandra Hospital Brisbane Australia; ^6^ Department of Medical Imaging Sunshine Coast University Hospital Birtinya Australia

**Keywords:** Education, Image interpretation, radiographers, randomised control trial

## Abstract

Radiographer commenting systems have not been successfully implemented in many Australian hospitals, despite evidence of their benefit and adoption elsewhere, such as the United Kingdom. An important contributor to the lack of widespread adoption of radiographer commenting in Australia (and likely elsewhere) is the limited availability of accessible education options for radiographers. The purpose of this randomised controlled trial is to compare the effectiveness of the same image interpretation education program delivered over an intensive 2‐day period (intensive format) versus a series of shorter regular workshops (non‐intensive format). The study design is a multicentre, stratified (by years of experience) two group parallel‐arm single‐blind (assessor blinded) randomised controlled trial. Participants will be allocated to one of the two groups: (1) intensive format of education or (2) non‐intensive format of education in a 1:1 ratio. Participants will undergo assessments before education, at 1 week post‐intervention completion and at 12 weeks post‐intervention completion. Findings from this trial will be of relevance to radiographers seeking image interpretation training as well as organisations providing image interpretation education to prepare clinical staff for participation in a radiographer commenting system. A limitation of the trial is that the sample will be inclusive of radiographers, and findings may not be able to be directly extrapolated to other clinical disciplines (e.g. junior doctors, physiotherapists or nurse practitioners).

## Background

Access to appropriate and timely medical imaging is important for the provision of effective emergency healthcare in hospital settings.^1^, ^2^ Findings from medical imaging investigations underpin many diagnostic and treatment decisions, particularly for people who have presented to hospital following a trauma event. Even with the rise in use of computed tomography since its development in 1972,^3^, ^4^ radiographic image series remains the primary imaging modality requested for patients who may have sustained trauma.^5^

In emergency settings, the definitive radiology report may not be available within a clinically relevant timeframe.^2^ This means that treatment decisions are likely to have been implemented by the referring clinical team before the radiologist's report is available in many cases. Unfortunately, delay between radiographic image capture and the availability of the radiologist's report can contribute to missed, incomplete or incorrect diagnoses.^2^, ^6^, ^7^ One approach to mitigate risk and better support junior or inexperienced members of the referring clinical team is the recording of a brief comment by the radiographer at the time of image capture to highlight any abnormalities they may have detected.^8^, ^9^, ^10^, ^11^

It has been suggested that radiographer commenting that highlights and describes acute abnormalities at the point of care may enhance patient management in hospital emergency departments.^10^, ^12^, ^13^, ^14^ However, this enhancement is dependent on radiographers' ability to detect and describe abnormalities when viewing and interpreting trauma radiographs. Importantly, radiographer performance in this regard can be improved as a result of targeted education in image interpretation.^15^, ^16^, ^17^, ^18^

A radiographer commenting system has not been successfully implemented in many Australian hospitals, despite evidence of its benefit and adoption elsewhere, such as the United Kingdom.^10^, ^13^, ^19^ An important contributor to the lack of widespread adoption of radiographer commenting in Australia (and likely elsewhere) may by the limited availability of accessible and effective training options for radiographers.^11^ Two key factors limiting access to appropriate education are likely to include the availability of suitably qualified experts to deliver image interpretation education and the ability of radiographers from geographically diverse locations to access it.

A potential solution to facilitate the accessibility of training is to offer different formats of education delivery that can be potentially flexible with radiographers' schedules. In preparation for the present trial, two specific formats for delivery of a short‐course image interpretation education program for radiographers were considered. The two formats include the same educational content delivered at different intensities by the same facilitators. One approach includes an intensive delivery 2‐day training suitable for radiographers who may require the training to be completed in a short amount of time (e.g. a rural radiographer travelling to a metropolitan centre or radiographers who can only complete training on days away from clinical work). The other approach is a non‐intensive delivery of short regular workshops that may have potential to be incorporated into regular hospital in‐service education programs. Although prior studies involving imaging interpretation among radiographers have been promising,^8^, ^9^, ^12^, ^16^ to date, there is no study that has compared the accuracy of radiographer image interpretation for skeletal X‐rays from radiographers who have received different formats of an educational intervention.

The purpose of this randomised controlled trial is to compare the effectiveness of the same image interpretation education program delivered over an intensive 2‐day period (intensive format) versus a series of shorter regular workshops (non‐intensive format). The intended effect of this education program is to improve radiographers' ability to detect and describe abnormalities visualised on trauma radiographs.

## Methods/Design

### Study design

**Figure 1:**
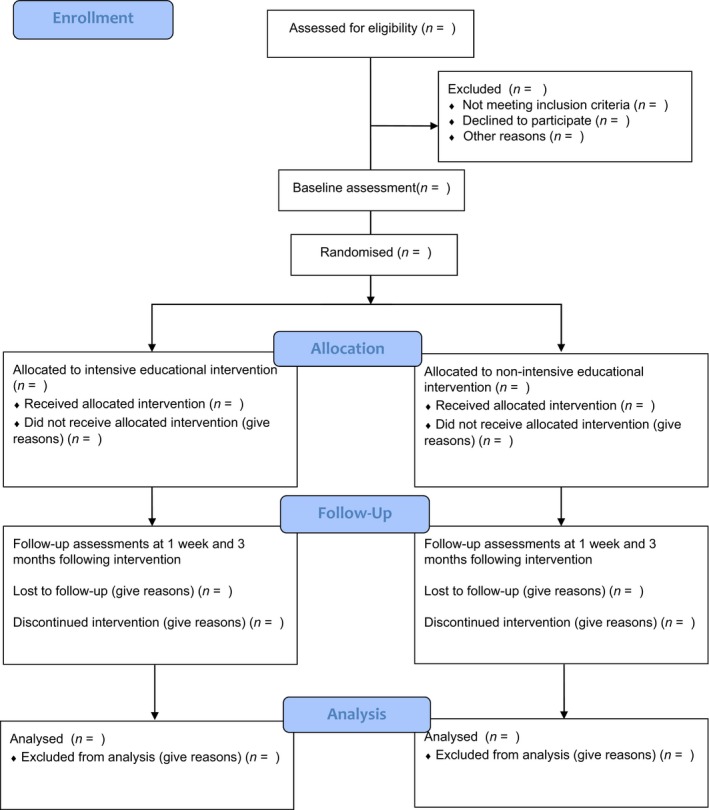
Study design – randomised control trial.

The Standard Protocol Items: Recommendations for Interventional Trials (SPIRIT) 2013 guidelines were used when preparing this study protocol.^20^ The study design is a multicentre, stratified (by years of experience) two group parallel‐arm single‐blind (assessor blinded) randomised controlled trial (Fig. 1). Participants will be allocated to one of the two groups: (1) intensive format of education or (2) non‐intensive format of education in a 1:1 ratio to examine whether either education delivery approach is superior to the other. Participants will undergo an assessment of their ability to interpret adult skeletal X‐rays, before education, at 1 week post‐intervention completion (primary time‐point) and at 12 weeks post‐intervention completion.

### Ethics statement

The Human Research Ethics Committees of both the Metro South Hospital and Health Service (HREC/11/QPAH/172) and the Queensland University of Technology (1200000061) approved this research.

### Setting and participant recruitment

Radiographers will be recruited from three metropolitan hospital medical imaging departments in southeast Queensland. These hospitals were chosen as they each have a dedicated emergency‐imaging department. Radiographers eligible for inclusion in the study must be currently working in an emergency‐imaging department and willing to undertake either intensive or non‐intensive image interpretation training. Radiographers in the participating sites will be invited to participate in the study via email invitation. This will be achieved by sending the radiographer clinical educator at each of the sites an email asking them to forward the invitation to participate to all their radiographers who rotate through their emergency‐medical imaging department. Radiographers will be excluded from the study if they: have previously completed formal education in image interpretation or commenting (e.g. a master's degree that included image interpretation coursework); are not available to attend training at the potential scheduled times; or do not hold full registration as a radiographer with the national board. Accompanying the original email invitation will be a participant information sheet and a consent form. The participant information sheet provides potential participants with the necessary understanding for the motivation and procedures that underpin this study as well as further information to allow them to give informed consent. There will also be opportunity for potential participants to contact a member of the research team for further information or to ask further questions. The participant information sheet also includes details explaining that the education sessions will be held out of normal business hours. For example, the intensive education will be held on weekends and the non‐intensive education will be held in the evenings. Participants are encouraged to ensure they are available to attend all the education sessions. Participants will provide written informed consent and are free to withdraw this consent at any time. Written consent forms will be collected by a member of the research team prior to participation.

### Randomisation

Computerised random number generation will be used to generate the randomisation sequence by a researcher not otherwise involved in the recruitment or assessment of participants. Participants will be stratified into four bands by years of clinical experience (1–2; 3–5; 6–12; 13 years+) to minimise the risk of differences in experience levels between groups. Concealment of allocation will occur through use of numbered opaque envelopes containing the group allocation for each participant and stored in a locked filing cabinet until the time of allocation. After a participant has completed their baseline assessment, an intervention co‐ordinator will be permitted to access the envelope with the corresponding participant number and open the envelope to reveal allocation (to either the intensive format or non‐intensive format). The baseline assessment will involve participants completing the primary and secondary outcome measures. This assessment will be conducted at the participant's place of work and scheduled in consultation with the participant and their manager to ensure their attendance.

### Intervention

The standardised education intervention will be delivered in two formats: one group will receive intensive education (2‐day intensive format of delivery) and the other group will receive non‐intensive or more traditional education (sequential 90‐min tutorials delivered (at least) 1 week apart).

Both formats contain identical content in the same overall ‘education time’ covering appendicular and axial skeletal trauma. The educational program was originally developed to enhance junior emergency doctors' skills in interpreting trauma radiographs. This program has been refined to suit radiographers by the original developer. To cater for multiple learning styles, the program incorporates a blended learning environment combining two distinct modes, passive traditional classroom teaching and an interactive visual practical component.^21^ Within the two modes of learning, a number of learning aids are included to cater for different learning styles incorporating multisense, psychomotor and affective strategies.^22^, ^23^ The education program also integrates several original acronyms and mnemonics to assist the learner with memory retention and support them practising the skills learnt to build capability.

The program is divided into nine 90‐min workshops (see Table 1) including using a search strategy; how to structure findings, common traumatic pathology, normal variants and frequently missed abnormalities. Paediatric trauma is not covered in this education program, as the education program has been developed at a site that is not a paediatric (<14 years of age) centre. To standardise intervention delivery, the same facilitators, both of whom are members of the research team, experienced in image interpretation and facilitation of training, will deliver the courses (together). The facilitators are neither aware of the contents of the assessments nor involved with marking the assessments. Class size will be comparable for both formats. To facilitate learning opportunities and interactions within the education sessions, the size of each class will be kept to approximately 12 students.^24^, ^25^

**Table 1:** Education intervention content outline

Workshop	Subject
1	General principles and strategy for interpretation of skeletal trauma
2	Hand, wrist and forearm
3	Face including mandible
4	Foot, ankle and tibia/fibula
5	Knee and distal femur
6	Pelvis and hips
7	Shoulder and humerus
8	Spine
9	Review of all content

To promote ongoing participation in the non‐intensive educational intervention, radiographers in this group will receive reminders prior to each weekly session via email. Furthermore, to promote completion of the education program for participants in both groups, this education program is eligible for accrual of continuing professional development points (equal to 13.5 h) recognised by the national registration board. A certificate of completion as evidence of accrual of the continuing professional development points will be provided to participants who complete the training.

### Outcome measures

#### Primary outcome measure

The primary outcome measure will be the Image Interpretation Test (IIT) assessment score, which has favourable evidence supporting its validity and reliability among radiographers.^26^ The development of this test has been described in detail in an earlier publication.^26^ This assessment involves participants examining a test bank of radiographic examinations (presented in random order) to try to identify abnormalities (and provide a descriptive comment when an abnormality is observed). The IIT assessment contains 60 examinations. It includes various appendicular and axial skeletal radiographs with a distribution of anatomical regions and proportion of abnormal cases that has been reported as consistent with a typical adult case mix from a hospital emergency department.^26^ This case mix was determined by conducting an audit of consecutive adult examinations over a 12‐month period. A description of this audit has been described previously.^26^ Participants in the trial will have their responses for each IIT examination (e.g. detection of normal or abnormal and description of abnormalities identified) scored by a blinded examiner. Two radiographers with postgraduate qualifications in image interpretation will serve as a panel of independent examiners to score and compare participant responses to the reference standard for each examination. The examiners will not be involved with the study design, conduct or education intervention (and blinded to group allocation and participant's identity). A reference standard has been created for each examination by a panel of experienced experts (two consultant radiologists and one consultant reporting radiographer).^26^ A novel rating scale has also been developed and validated in an earlier publication (Table 2).^26^ The examiners will be trained on how to use the rating scale by the site investigator (MN). They will be provided with a guide and worksheet to enable a consistent framework for marking. By using the rating scale, each examination in the IIT will be given a numerical value with a maximum total score of 3 and a minimum of 0. When marking assessments, discordant ratings will be discussed between the examiners until consensus reached.

**Table 2:** Scoring criteria for each examination in the Image Interpretation Test

Criteria	Score
For radiographic cases with a traumatic abnormality	
Abnormality not detected	0
Abnormality detected, but not described correctly	1
Abnormality detected, description incomplete (but not incorrect)	2
Abnormality detected and correctly described in entirety	3
For radiographic cases with no traumatic abnormality	
False abnormality reported or described	0
Correct report of absence of any traumatic abnormality	3

To determine which format of delivery resulted in greater improvement and maintenance of image interpretation capability, the IIT will be completed prior to education commencement, 1 week after education completion and at 12 weeks post‐education completion for both formats. A 12‐week reassessment will provide an indication of whether there is difference in the maintenance of improvements observed at the first post‐intervention assessment. The 12‐week duration was also chosen so that a comparison could be made between the 12‐week post‐intensive format assessment, with the 1‐week post‐non‐intensive format assessment as these would be approximately the same number of weeks post‐intervention commencement (i.e. approximately 13 weeks). To help limit case recall bias at individual assessment points, each test bank of images will be presented in random order. The order of the cases will be determined by a computer‐generated randomisation sequence. To minimise the impact of post‐test discussion, participants will be asked not to discuss the cases within the test. This however could not be directly monitored or controlled by the investigators.

#### Primary outcome measure procedure

Before each assessment, radiographers will be provided with a guideline (see Table 3) for classification of radiographic examinations based upon a prior investigation.^27^ The anonymised DICOM (Digital Imaging and Communications in Medicine) images will be embedded into an image review software program (Codonics Clarity Viewer version 6.1, Middleburg Heights, Ohio, USA). Prior to interpreting and commenting on radiographs in the IIT, each participant will receive instruction on how to use this software to view each image. This software program has the capability to adjust image contrast/density, zoom, pan and invert an image as would be possible in clinical practice settings.

**Table 3:** Examination classifications for primary outcome measure

Findings
Abnormal
1. Fractures
2. Joint disruptions
3. Joint effusions
4. Soft tissue swelling
Normal
1. Anatomical variants
2. Non‐traumatic pathology
3. Old fractures
4. Evidence of previous surgery

To simulate the clinical setting and typical workflow, the assessment will take place in a semi‐darkened room with a time restriction (a total of 90 min assigned to complete the assessment). The images will be reviewed using a standard personal computer monitor (2‐megapixels) that is consistent to reviewing images in the clinical setting. Neither a clinical history nor access to previous imaging for each examination in the IIT will be permitted. Not including clinical history is designed to ensure participant's responses to the IIT are entirely dependent on how well they scan and interpret the radiographic images, rather than an assessment of how well they interpret case histories in combination with radiographic images.

#### Secondary outcome measure

In addition to providing a description of the pathology (perceived to be) present for each examination, the participants will be asked to provide a ‘confidence rating’ to indicate how confident they are in their interpretation on a 5‐point Likert scale (normal, probably normal, possibly normal, probably abnormal and abnormal). These ratings will be reported at baseline and at follow‐up assessments.

Two questionnaires will also be administered (the first to be completed at the baseline assessment prior to randomisation, the second is completed at the 12‐week post‐intervention assessment). The content and format of the two questionnaires is comparable, with the exception of an additional question in the second questionnaire to investigate the participant's perception of the education format they received. These questionnaires will be completed via a secure web‐based platform. The development process for these secondary questionnaires followed recommendations for the design and conduct of self‐administered surveys for clinicians.^28^ The process involved forming a panel of local content and survey design experts who assisted in item generation, reduction and pretesting. Both electronic questionnaires were piloted with a small convenience sample of radiographers (*n* = 6). Questionnaire flow, format, interpretability, redundant items and overall length of questionnaires were assessed. Each questionnaire will be distributed to all participants via an email containing a hyperlink to the secure web‐based survey platform.

#### Sample size calculation

Owing to a lack of similar studies to inform effect size assumptions, the research team took a pragmatic approach to determining an appropriate effect size to use in the sample size calculation.^29^ The team consulted three medical imaging clinical educators to consider what they would classify as a meaningful difference in the primary outcome measure that would lead them to choose one education delivery format over the other. Their responses indicated that a difference of more than one correctly described image, which equated to 4 points or more on the IIT, would be considered a significant difference. Therefore, the target sample size will be 48 (24 participants per group). A sample size of 24 participants per group provides greater than 80% power^29^ to detect a 4‐point difference between groups in the primary outcome (IIT score) at a significance level of 0.05%, assuming a standard deviation of 4.5 and a dropout rate <15%.

### Statistical methods

Data analyses will be performed using Stata (StataCorp, College Station, TX, USA). Conventional descriptive statistics will be used to describe the sample (years experience as a radiographer, age, gender, current or previous involvement in a radiographer abnormality detection system, as well as the primary and secondary outcomes). Data will be analysed according to the intention‐to‐treat principles. Outcome measures will be compared within and between intervention groups at baseline, and follow‐up assessments using generalised linear models to determine whether there were differences between groups and changes in primary or secondary outcomes over time (group by time interaction). The primary time‐point of interest is the 1‐week post‐intervention assessment. From these analyses, it will be possible to determine which method of education delivery (if any) had a greater impact on improving the radiographers' ability to detect and describe abnormalities on trauma radiographs, with the opportunity to adjust for baseline confounders if indicated.

## Discussion

Errors in the interpretation of radiographs in the emergency department have important consequences for patients and clinicians.^5^ Failure to correctly identify abnormalities on radiographs in a timely manner may result in delayed or inappropriate treatment leading to suboptimal outcomes for patients and potential medicolegal claims against clinicians.^6^ Radiographer commenting has been proposed as a mechanism to reduce diagnostic errors by providing an immediate opinion on a radiograph at the time of image capture by the performing radiographer.^10^, ^12^, ^14^ The potential benefits of radiographer commenting are likely to be dependent on the radiographer's ability to detect and describe abnormalities when viewing and interpreting trauma radiographs. Consequently, access to effective training that radiographers receive in preparation for a radiographer commenting role is paramount. This will be the first randomised controlled trial to evaluate the effectiveness of intensive and non‐intensive formats of delivery of image interpretation education. This study will generate valuable evidence regarding how image interpretation education can be effectively delivered to radiographers in hospital settings nationally and internationally.

There are several limitations of this study that should be considered. The possibility of recall bias is always a potential limitation with studies that involve the completion of the same assessment during a relatively short time frame. To minimise participants memorising cases between assessments, this study will undertake two preventative actions. First, a minimum time period between any two assessments of 5 weeks will be employed. Second, a computer‐generated randomisation sequence will be used to present each 60 case assessment in random order, further limiting the potential for case recall. An additional limitation that is a risk when employing a voluntary sampling method for participation in a research study is the possibility of self‐selection bias. Radiographers who respond to the participation invite may have a greater interest in image interpretation education than non‐responders. Another potential limitation is that there was no pilot data to inform sample size calculations prior to the study commencement. If significant findings are not observed in the final analyses, a post‐hoc power calculation based on trial data will be conducted to inform planning for future studies. Furthermore, the knowledge and capabilities of rural radiographers in comparison to radiographers from metropolitan areas has not been contemplated in this study and may be considered a limitation of this trial which required participants to be available to receive either intervention format. Future research investigating their difference would be worthwhile.

Findings from this trial are likely to be of relevance to radiographers seeking image interpretation training as well as organisations providing image interpretation education to prepare clinical staff for participation in a radiographer commenting system. A further limitation of the trial is that the sample will be inclusive of radiographers, and findings may not be able to be directly extrapolated to other clinical disciplines (e.g. junior doctors, physiotherapists or nurse practitioners). Nonetheless, this trial will provide insight into how image interpretation education can be effectively delivered and pave the way for future research among other relevant health professions including physiotherapists, nurse practitioners and doctors who work in emergency department settings.

## Trial status

**Trial Registration:** Australian New Zealand Clinical Trials Registry – ACTRN12612000210875. The trial was recruiting at the time of submission.

## Conflict of Interest

The authors declare no conflict of interest.
